# Lower activity of cholesteryl ester transfer protein (CETP) and the risk of dementia: a Mendelian randomization analysis

**DOI:** 10.1186/s13195-024-01594-6

**Published:** 2024-10-16

**Authors:** Amand F. Schmidt, Michael H. Davidson, Marc Ditmarsch, John J. Kastelein, Chris Finan

**Affiliations:** 1 Institute of Cardiovascular Science, Faculty of Population Health, University College London, 69-75 Chenies Mews, London, WC1E 6HX UK; 2 UCL British Heart Foundation Research Accelerator, 69-75 Chenies Mews, London, WC1E 6HX UK; 3 Department of Cardiology, Amsterdam Cardiovascular Sciences, Amsterdam University Medical Centres, University of Amsterdam, Amsterdam UMC, locatie AMC Postbus 22660, Amsterdam Zuidoost, 1100 DD The Netherlands; 4 Department of Cardiology, Division Heart and Lungs, University Medical Center Utrecht, Utrecht University, Heidelberglaan 100, Utrecht, 3584 CX The Netherlands; 5 Pritzker School of Medicine, University of Chicago, 5801 S Ellis Ave, Chicago, IL 60637 USA; 6 NewAmsterdam Pharma B.V, Gooimeer 2-35, Naarden, 1411 DC Netherlands; 7 Department of Vascular Medicine, Amsterdam Cardiovascular Sciences, Amsterdam University Medical Centres, University of Amsterdam, Amsterdam UMC, locatie AMC Postbus 22660, Amsterdam Zuidoost, 1100 DD The Netherlands

**Keywords:** Cholesteryl ester transfer protein, Dementia, *APOE4*, Lewy body dementia, Dementia in Parkinson, Mendelian randomisation, Human genetics

## Abstract

**Background:**

Elevated concentrations of low-density lipoprotein cholesterol (LDL-C) are linked to dementia risk, and conversely, increased plasma concentrations of high-density lipoprotein cholesterol (HDL-C) and apolipoprotein-A1 (Apo-A1) associate with decreased dementia risk. Inhibition of cholesteryl ester transfer protein (CETP) meaningfully affects the concentrations of these blood lipids and may therefore provide an opportunity to treat dementia.

**Methods:**

Drug target Mendelian randomization (MR) was employed to anticipate the on-target effects of lower CETP concentration (μg/mL) on plasma lipids, cardiovascular disease outcomes, autopsy confirmed Lewy body dementia (LBD), as well as Parkinson’s dementia.

**Results:**

MR analysis of lower CETP concentration recapitulated the blood lipid effects observed in clinical trials of CETP-inhibitors, as well as protective effects on coronary heart disease (odds ratio (OR) 0.92, 95% confidence interval (CI) 0.89; 0.96), heart failure, abdominal aortic aneurysm, any stroke, ischemic stroke, and small vessel stroke (0.90, 95%CI 0.85; 0.96). Consideration of dementia related traits indicated that lower CETP concentrations were associated higher total brain volume (0.04 per standard deviation, 95%CI 0.02; 0.06), lower risk of LBD (OR 0.81, 95%CI 0.74; 0.89) and Parkinson’s dementia risk (OR 0.26, 95%CI 0.14; 0.48). *APOE4* stratified analyses suggested the LBD effect was most pronounced in *APOE-*ε4 + participants (OR 0.61 95%CI 0.51; 0.73), compared to *APOE-*ε4- (OR 0.89 95%CI 0.79; 1.01); interaction *p*-value 5.81 × 10^− 4^.

**Conclusions:**

These results suggest that inhibition of CETP may be a viable strategy to treat dementia, with a more pronounced effect expected in *APOE-*ε4 carriers.

**Supplementary Information:**

The online version contains supplementary material available at 10.1186/s13195-024-01594-6.

## Background

Cholesteryl ester transfer protein (CETP) facilitates the exchange of triglycerides (TG) and cholesterol ester between high-density lipoprotein cholesterol (HDL-C) and apolipoprotein-B (Apo-B) rich particles such as low-density lipoprotein cholesterol (LDL-C). CETP-inhibition has shown to elicit a plethora of beneficial effects on lipid metabolism, robustly decreasing the plasma concentration of canonical atherosclerosis particles such as total and small LDL, and lipoprotein (a) (Lp[a]), while increasing plasma concentrations of mature HDL, as well as pre-beta HDL and apolipoprotein-A1 (Apo-A1) [1–4].

The four CETP-inhibitors (CETPi) evaluated in phase 3 clinical trials (anacetrapib, evacetrapib, dalcetrapib, torcetrapib) showed heterogenous effects on the magnitude of lipid perturbation, with an HDL-C percentage increase between 29% for dalcetrapib and ∼ 130% for anacetrapib/evacetrapib, and an LDL-C decrease between 1% for dalcetrapib and 20% for anacetrapib/evacetrapib [5]. This resulted in an equally mixed clinical effects profile [5], with only the REVEAL trial for anacetrapib showing a non-HDL-C proportional protective effect of CETPi on CVD onset (rate ratio 0.91; 95% confidence interval (95%CI) 0.85; 0.97) [6]. The presence of meaningful differences in clinical effects profile strongly suggests that previous CETPi failures are likely attributable to the specific compound rather than to CETP inhibitors as a class [7].

We have previously determined the viability of a reduction in CETP concentration using Mendelian randomization (MR), leveraging genetic instruments strongly associating with plasma CETP concentration, finding that lower plasma CETP concentration decreased the risk of CHD, heart failure (HF) and chronic kidney disease [5, 8]. Because genetic variants are protected against confounding bias and reverse causation, MR provides a robust indication of the likely on-target effects of sufficiently potent drug target perturbation using data from human subjects [9–11]. A further benefit of MR is that it can utilize aggregated genetic data (e.g., variant-specific point estimates and standard errors) from independent studies to maximize the available sample size and hence precision.

Given the robust LDL-C lowering effects of CETP-inhibition, research has understandably focussed on its potential implications for cardiovascular disease (CVD) prevention. However, all CETP inhibitors, including the novel CETP-inhibitor obicetrapib, increase plasma concentrations of apolipoprotein-E (Apo-E) [4], which is associated with decreased risk of dementia, in particular for Alzheimer’s disease (AD). Furthermore, the 2024 Lancet Commissions report determined that LDL-C is a potentially modifiable risk factor for dementia [12]. Multiple lines of evidence support involvement of lipid metabolism with bioenergetic decline and chronic neuro-inflammation in the brain, which contributes to neurodegenerative disorders [13]. This interrelationship between metabolism and neurodegeneration is for example illustrated by the connection between amyloid-β, Apo-E isoforms and lipid trafficking associating with the onset dementias such as AD, Lewy body dementia (LBD), and dementia associated with Parkinson’s Disease (PD) [14–17]. Noting that LBD and dementia in PD are closely related diseases, both caused by underlying Lewy body disorders, which predominantly differ in temporal sequence of symptoms and clinical features [18].

The Apo-E isoform Apo-E4 is a major determinant of AD risk, with homozygote carriers (*APOE-*ε4ε4) conveying an up to 15 fold increased risk [19, 20]. Through effects on neuroinflammation and blood brain barrier integrity *APOE-*ε4 carriership is an important risk factor for AD and non-AD related dementias [21, 22]. AD in *APOE-*ε4 carriers [13] is characterized by higher levels of circulating tau, as well as accumulation of phosphorylated tau in brain, which has been associated with insufficient lipidation of Apo-E HDL particles [23]. Lack of particle lipidation dysregulates the fine balance between cholesterol availability to neurons and cholesterol accumulation in astrocytes, which has cytotoxic and proinflammatory consequences [24]. The lack of lipidation of Apo-E/HDL additionally affects astrocyte membrane composition, stimulating the formation of β-amyloid containing plaques, which is a major characteristic of AD brains. *APOE-*ε4 carriership and cholesterol metabolism has additionally been implicated in the development amyotrophic lateral sclerosis (ALS) as well as multiple sclerosis (MS) [25–27].

Given the central role of CETP in lipid metabolism and the fact that CETP inhibition influences HDL composition of Apo-E [28] as well as brain cholesterol concentration [29], the CETP inhibitor obicetrapib is now being considered for treatment of dementia which is tested in a large phase II clinical trial (BROADWAY). Using multivariable MR, conditioning on LDL-C concentration, we previously revealed an HDL-C mediated protective effect of lower CETP on AD: odds ratio (OR) 0.94 per SD increase in HDL-C (95%CI 0.89; 0.99) [5]. While the observed HDL-C mediated effect of CETP on AD closely follows the aforementioned relationship of Apo-E HDL particles and their role in AD, likely due to the absence of *APOE4* stratification and robust AD case ascertainment, the main univariable MR analysis of CETP concentration and AD did not reach similar statistical significance (OR 0.99, 95%CI 0.91; 1.07).

In the current study we therefore sought to elucidate the potential causal relationship between lower plasma CETP concentration and the risk of dementia and neurodegenerative diseases. Specifically, we considered independent GWAS on any LBD, LBD stratified by *APOE-*ε4 status, PD, dementia in PD – representing a disease clustering with a strong *APOE-*ε4 contribution. Furthermore, to rule out potential disease miss-classification, which is common in dementia, we uniquely included autopsy confirmed LBD cases and controls [30, 31]. As a positive control we first sought to confirm our previously reported effects on CHD, expanding this to additional CVD outcomes including small vessel stroke, and abdominal aortic aneurysm (AAA). Finally, we sought to replicate associations with biomarker levels and disease onset by performing additional MR analyses weighting the *cis*-acting CETP variants by their association with Apo-A1, and Apo-B (downstream proxies of CETP activity). Importantly, as shown by Schmidt et al. [9, 11] a *cis*-MR analysis weighted by downstream effects of the protein does not require, or imply, that the weighting factor itself causes disease. Instead, the weights merely function as a proxy for protein value and activity. Hence inference in these Apo-A1 and Apo-B weighted analyses remains on the effect of lower CETP and does not address questions on potential lipoprotein mediation.

## Methods

### Selection of genetic instruments to model CETP effects

Genetic instruments associating with CETP concentration (μg/mL) were identified from a GWAS conducted by Blauw et al. [32] (*n* = 4,248). To limit the potential for bias-inducing pre-translational horizontal pleiotropy [9, 11] we applied a *cis* window of ± 25 kilobase pair (kbp) around *CETP* (ENSG00000087237, GRCh37), noting that this includes the entire GWAS signal observed in the source GWAS. Variants were selected to have an F-statistic of 24 or larger, and a minor allele frequency (MAF) of 0.01 or larger. The F-statistic threshold was used to limit the potential influence of weak-instrument bias [33]. Through our two-sample design any potential remaining weak-instrument bias is expected to act towards a neutral effect direction, guarding against an increased false positive rate. The MAF threshold was chosen to ensure we could robustly model genetic linkage disequilibrium (LD) [9] based on a random sample of 5,000 UK biobank participants as a reference. Using these references data, the genetic variants were clumped to an R-squared of 0.30, using the same reference data to model the residual LD (see below).

As described in Schmidt et al. 2020 [9] genetic associations with downstream consequences of protein expression can be used as an additional source of instrument selection and modelling using Mendelian randomization (MR). This provides opportunities to replicate the results observed in *cis*-MR using genetic associations with protein concentration. MR analyses using downstream proxies of protein expression reflect effects of protein activity, complementing analyses of protein concentration. Here we used GWAS on plasma concentration of Apo-A1 and Apo-B (gwas.mrcieu.ac.uk, study ID: met-d, n: 111,078) from which we extracted genetic variants, applying the same variant selection criteria centred on the *cis*-CETP region. We differentiate between the three analysis by referring to MR analyses weighted by “CETP”, “Apo-A1”, or “Apo-B”. Similarly, when utilising CETP variants from the Blauw et al. GWAS on CETP plasma concentration, we refer to these MR effects as the effect of lower CETP concentration, reflecting that under the core instrumental variable assumptions [34] the effects of genetically predicted CETP concentration is equivalent to the effects of CETP concentration itself.

### Genetic sources of outcome data

Using MR, the identified CETP instruments were related to GWAS data on the following traits: plasma concentrations of Apo-A1, Apo-B, intermediate-density lipoprotein cholesterol (IDL-C), very-low-density lipoprotein cholesterol (VLDL-C), remnant-C from (https://gwas.mrcieu.ac.uk/datasets, dataset: met-d, n: 111,078), LDL-C, HDL-C, and TG from [35] (n: 1,320,016), Lp[a] from (n: 361,194, http://www.nealelab.is/uk-biobank), systolic/diastolic blood pressure (SBP/DBP) from [36] (n: 757,601), brain volume from [37] (n: 47,316), white matter hyperintensity volume from [38] (WHM vol., n: 42,310, https://www.ebi.ac.uk/gwas/publications/32358547), circulation total tau from [39] (Circ. total tau, n: 14,721), CHD from [40] (cases: 181,522, total n: 1,165,690), any stroke, ischemic stroke, small vessel stroke from [41] (any stroke cases: 110,182, total n: 1,614,080; any ischemic stroke cases: 86,668, total n: 1,590,566; small vessel stroke cases: 9,219, total n: 1,517,518), atrial fibrillation from [42] (AF, cases: 60,620, total n: 1,030,836), HF from [43] (cases: 115,150, total n: 1,665,481), AAA (cases: 8,163, total n: 1,164,713, https://www.globalbiobankmeta.org/), LBD from [30] (cases: 2,981, total n: 6,618), LBD stratified on *APOE-*ε4 status from [31] (positive cases: 1,180, positive total n: 1,837, negative cases: 1,286, negative total cases: 3,557), PD from [44] (cases: 56,306, total n: 14,056,306), dementia in PD from [45] (cases: 263, total n: 3,923), multiple sclerosis from [46] (cases: 14,498, total n: 38,589), amyotrophic lateral sclerosis from [47] (cases: 15,156, total n: 41,398).

### Mendelian randomization analysis

*Cis-*MR was employed to ascertain the possible causal effects of low CETP concentration on neurodegenerative disease and cardiovascular outcomes. MR estimates were calculated using generalized least squares (GLS) implementations of the inverse-variance weighted (IVW) estimator and the MR-Egger estimator, the latter being unbiased in the presence of horizontal pleiotropy at the cost of lower precision. We used GLS to directly model the LD reference structure, after clumping to an R-squared of 0.30, optimizing power while preventing potential multicollinearity-based numerical instability. To minimize the potential influence of horizontal pleiotropy, variants beyond 3 times the mean leverage or with an outlier (Chi-square) statistic larger than 10.83, were pruned [48]. Finally, a model selection framework was applied to select the most appropriate estimator, IVW or MR-Egger [48, 49]. This model selection framework [50] utilizes the difference in heterogeneity between the IVW Q-statistic and the Egger Q-statistic to decide which method provides the best model to describe the available data and hence optimizes the bias-variance trade-off.

### Effect estimates and multiple testing

Effect estimates are presented in the CETP lowering direction, for the CETP and Apo-B weighted MR analyses this implies the results are presented towards the decreasing direction, while the Apo-A1 weighted analyses are presented in the increasing direction. Results are provided with 95% confidence intervals (CI) and *p*-values. Statistical significance was determined by comparing the *p*-values against a multiplicity corrected threshold of 0.05/29 ≈ 1.7 × 10^− 3^ for the main analysis focussing on associations with biomarkers and disease. Furthermore, results of the main analysis were replicated by identifying significant and directionally concordant results using the *cis*-MR analysis of CETP activity weighted by Apo-A1 concentration and Apo-B concentration.

## Results

*Cis*-MR was employed to evaluate the potential causal effects lower CETP had on biomarker and disease traits. Specifically, we sourced instruments from a ± 25 kbp window within and around *CETP* (ENSG00000087237), selecting variants based on GWAS’ of CETP concentration (no. participants: 4,248), Apo-A1 concentration (no. participants: 355,729), or Apo-B concentration (no. participants: 355,729).

### Effects of lower CETP concentration on biomarker traits

**Fig. 1:**
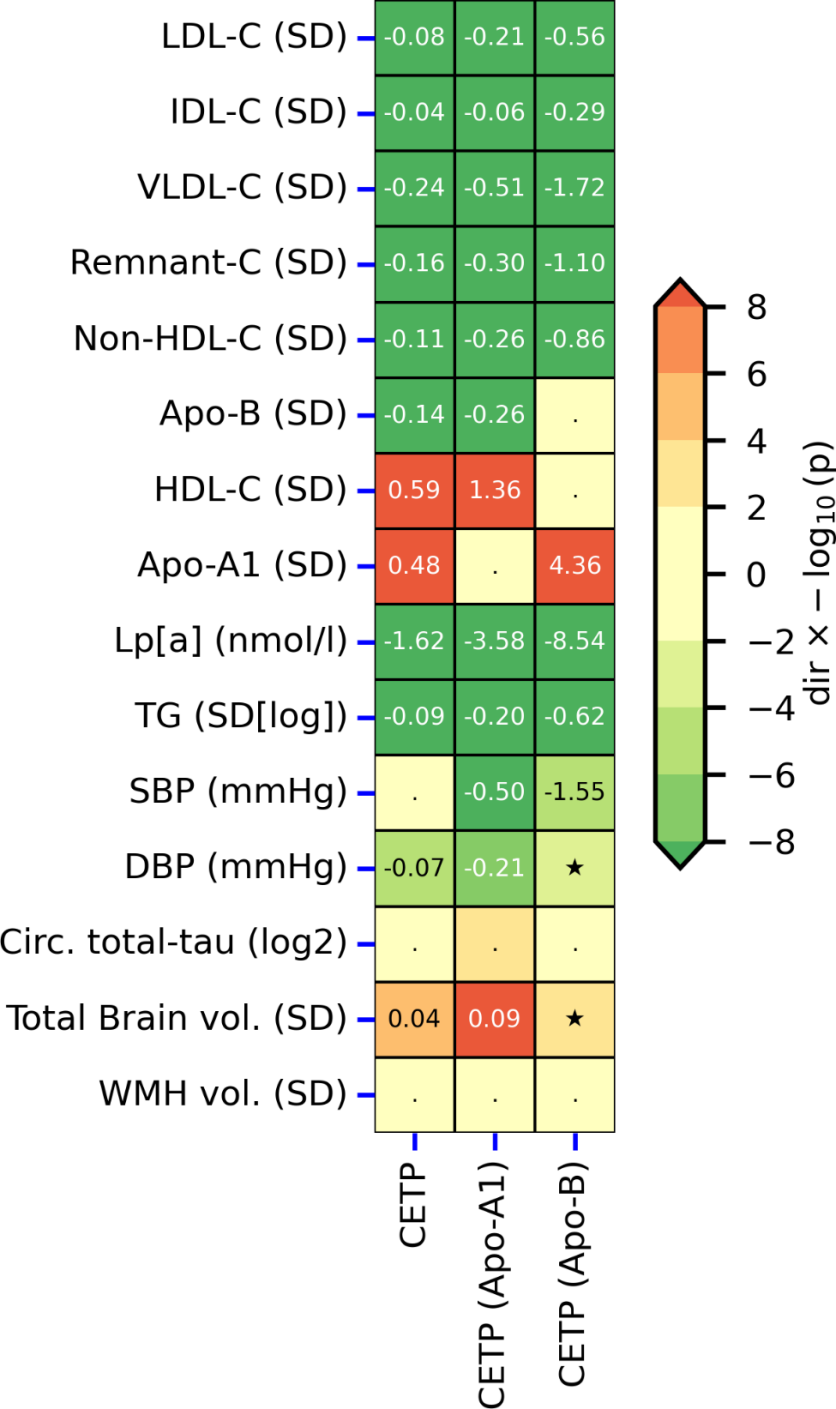
Biomarker effects of lower CETP level/activity estimated through *cis* Mendelian randomization using three distinct weighting strategies The MR effects were estimated by alternatingly selecting instruments based on the genetic association with lower CETP concentration (μg/mL), higher Apolipoprotein-A1 (Apo-A1 in g/L), and lower Apolipoprotein-B (Apo- B in g/L). Results are presented as effect direction multiplied by the -log10(*p*-value), truncated to a maximum of 8. Results with a *p*-value smaller than 0.05/29 are annotated by the point estimates rounded to two decimal places, nominal significance with a *p*-value between 0.05 and 0.05/29 is indicated by a star symbol, with results above 0.05 indicated by a dot. For the Apo-A1 weighted analyses, the Apo-A1 association was removed (reflecting identical data), with similar masking for the Apo-B weighted analysis. The outcome traits are listed on the y-axis with their units in brackets. Abbreviations: LDL-C, low-density lipoprotein cholesterol; IDL-C, intermediate-density lipoprotein cholesterol; VLDL-C, very low-density lipoprotein cholesterol; Remnant-C, remnant cholesterol, non-HDL-C, non-high-density lipoprotein cholesterol, HDL-C, high-density lipoprotein cholesterol; Lp[a], lipoprotein a; TG, total triglycerides; SBP, systolic blood pressure; DBP, diastolic blood pressure; WMH, white matter hyperintensity; vol., volume, Circ. circulating. Please see Supplementary Table S1 for the full results, and Table S2 for the source data and sample size

Lower CETP concentration (Fig. 1, Table S1-S2) was associated with a decrease in plasma concentration of LDL-C (-0.082 standard deviation (SD), 95%CI -0.086; -0.079), IDL-C (-0.04 SD, 95%CI -0.05; -0.03), VLDL-C (-0.24 SD, 95%CI -0.27; -0.21), remnant-cholesterol (-0.16 SD, 95%CI -0.17; -0.15), non-HDL-C (-0.113 SD, 95%CI -0.119; -0.107), TG (-0.085 SD, 95%CI -0.090; -0.081), Apo-B (-0.144 SD, 95%CI -0.153; -0.135), and Lp[a] (-1.62 nmol/L, 95%CI -1.91; -1.32). Following the canonical CETPi effects, genetically instrumented lower CETP increased the concentration of HDL-C (0.59 SD, 95%CI 0.57; 0.60), and Apo-A1 (0.48 SD, 95%CI 0.45; 0.52), respectively.

CETP additionally affected non-lipid traits, where lower CETP concentration decreased DBP − 0.07 mmHg (95%CI -0.11; -0.03), and increased total brain volume 0.04 SD (95%CI 0.02; 0.06), respectively. The presented results were replicated in MR analyses selecting and weighting *CETP* genetic instruments by Apo-A1 and/or Apo-B concentration as a proxy for reduced CETP activity; Fig. 1.

### Effects of lower CETP concentration on cardiovascular outcomes

**Fig. 2:**
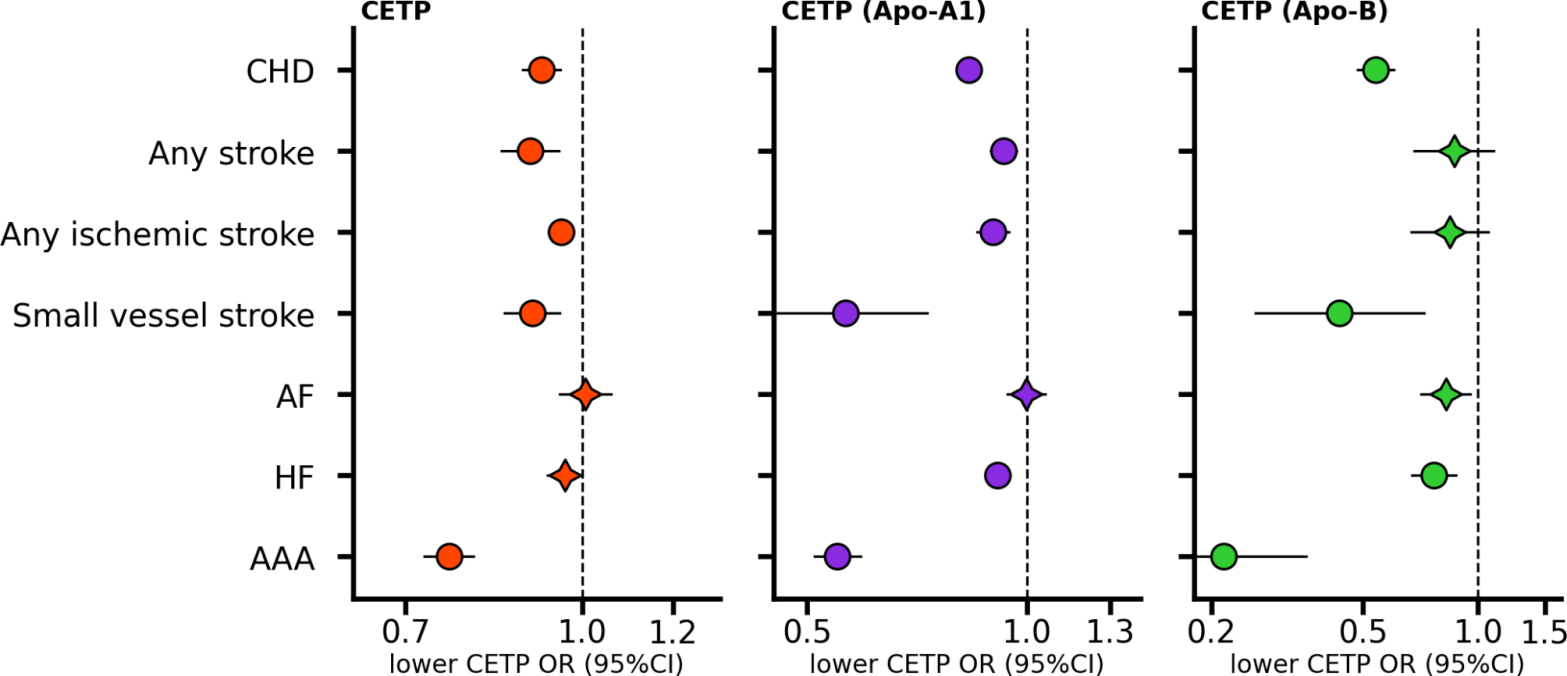
Cardiovascular effects of lower CETP level/activity on cardiovascular disease estimated through *cis* Mendelian randomization using three distinct weighting strategies The MR effects are estimated by alternatingly selecting instruments based on the genetic association with lower CETP concentration (μg/mL), higher Apolipoprotein-A1 (Apo-A1 in g/L), and lower Apolipoprotein-B (Apo- B in g/L). The estimated odds ratio (OR) is indicated by a circle if the *p*-value was smaller than 0.05/29, or by a star otherwise, with the horizontal bars representing 95% confidence intervals (95%CI), a neutral effect of 1 is indicated by the dashed vertical line. Abbreviations: CHD, coronary heart disease; AF, atrial fibrillation; HF, heart failure; AAA, abdominal aortic aneurysm. Please see Supplementary Table S1 for the full results, and Table S2 for the source data and sample size

We confirmed that lower plasma CETP concentration decreased the risk of CHD (OR 0.92, 95%CI 0.89; 0.96, *p*-value 2.47 × 10⁻⁵), any stroke (OR 0.90, 95%CI 0.85; 0.95), any ischemic stroke (OR 0.96, 95%CI 0.94; 0.98), as well as small vessel stroke (OR 0.90, 95%CI 0.85; 0.96); Fig. 2, Table S1. We additionally observed that lower plasma concentration of CETP decreased the risk of AAA (OR 0.76, 95%CI 0.73; 0.80) and HF (OR 0.97, 95%CI 0.93; 1.00, *p*-value 4.39 × 10⁻²), although the latter only reached nominal significance. Aside from the any stroke and ischemic stroke signals, which were partially replicated by Apo-A1 weighted MRs, the associations with CHD, small vessel stroke, HF and AAA were fully replicated by *cis*-MR analyses selecting and weighting *CETP* variants by their associations on Apo-A1 or Apo-B concentration; Fig. 2.

### Effects of lower CETP concentration on neurodegenerative outcomes

**Fig. 3:**
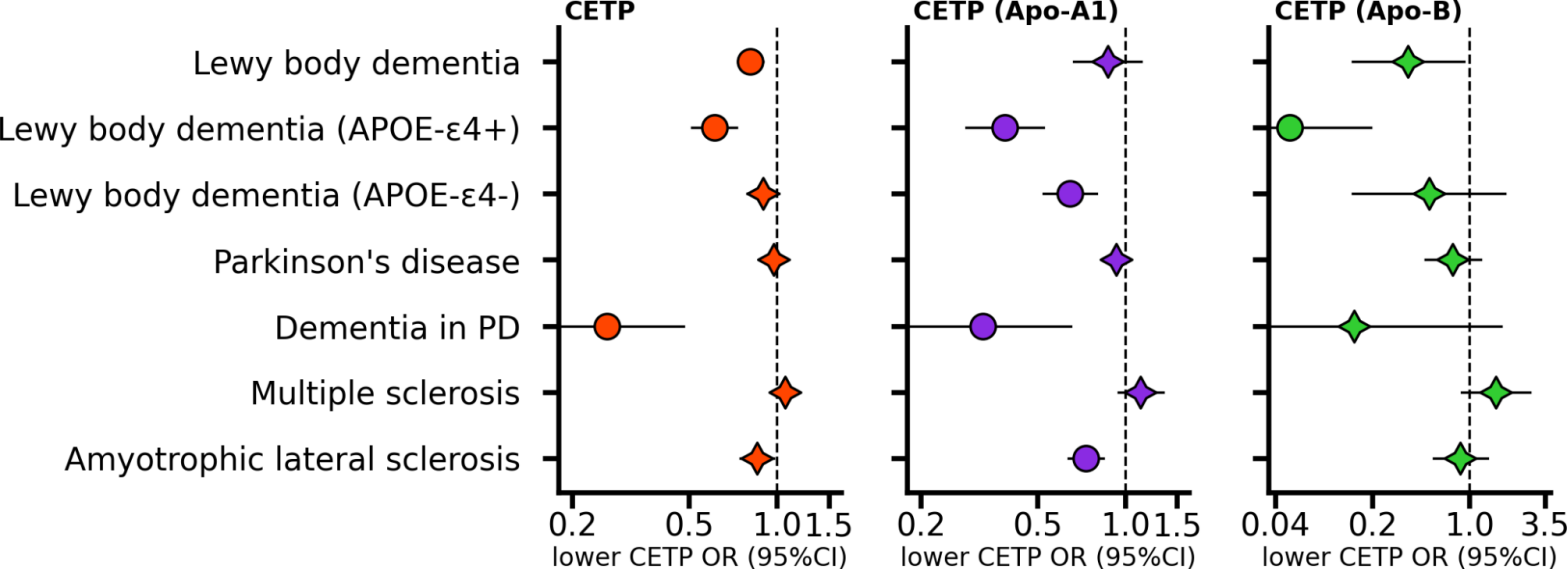
Effects of lower CETP level/activity on neurological traits estimated through *cis* Mendelian randomization using three distinct weighting strategies The MR effects are estimated by alternatingly selecting instruments based on the genetic association with lower CETP concentration (μg/mL), higher Apolipoprotein-A1 (Apo-A1 in g/L), and lower Apolipoprotein-B (Apo- B in g/L). The estimated odds ratio (OR) is indicated by a circle if the *p*-value was smaller than 0.05/29, or by a star otherwise, with the horizontal bars representing 95% confidence intervals (95%CI), a neutral effect of 1 is indicated by the dashed vertical line. Abbreviations: PD, Parkinson’s disease; *APOE-*ε4 + refers to carriers, *APOE-*ε4- refers to non-carriers. Please see Supplementary Table S1 for the full results, and Table S2 for the source data and sample size

Noting that lower plasma concentration of CETP associated with higher brain volume, we next explored associations with neurodegenerative traits. We found that lower CETP concentration was associated with a decrease in LBD (OR 0.81, 95%CI 0.74; 0.89, *p*-value 2.95 × 10⁻⁵), where *APOE4-* ε4 status modified this association: LBD in *APOE-*ε4 carriers (OR 0.61, 95%CI 0.51; 0.73, *p*-value 4.91 × 10⁻⁸), and non *APOE-*ε4 carriers (OR 0.89, 95%CI 0.79; 1.01, *p*-value 8.06 × 10⁻²); interaction *p*-value 5.81 × 10^− 4^. We further observed that lower CETP concentration protected against dementia in Parkinson’s disease (OR 0.26, 95%CI 0.14; 0.48, *p*-value 1.29 × 10⁻⁵), which partially overlaps with known LBD pathophysiology; Fig. 3, Table S1. Additionally, we observed a nominal risk decreasing effect of lower CETP on ALS (OR 0.85, 95%CI 0.75; 0.97, *p*-value 1.64 × 10⁻²). The apolipoprotein (both Apo-A1 and Apo-B) weighted analyses replicated the effect on LBD in *APOE-*ε4 carriers, with the Apo-A1 weighted analysis also replicating the associations for dementia in PD, as well as the ALS association; Fig. 3.

## Discussion

In the current analysis we employed *cis*-MR to determine biological consequences of lower CETP activity. We recapitulated and extended known beneficial effects of lower CETP levels on blood lipids, as well as protective effects on cardiovascular diseases such as CHD, AAA, HF, and small vessel stroke. Subsequently we explored potential associations with dementia related traits, noting protective effects of lower CETP concentrations with autopsy confirmed LBD, which was most pronounced in *APOE-*ε4 carriers – compatible with the previously observed protective effect of loss-of-function CETP variants in *APOE-*ε4 carriers [51]. The effects of lower CETP concentration were replicated by performing *cis*-MR weighting *CETP* variants by their association on Apo-A1 and Apo-B concentration.

The observed association between low activity of CETP and LBD is in-line with our previous observations that CETP has an HDL-C mediated effect on AD. These findings are also supported by our current understanding about the role of lipid metabolism in dementia, where increased concentrations of HDL particles and Apo-A1, may offer an *APOE*-ε4 dependent effect on cholesterol transport, and clearance of oxysterol and β-amyloid. It is worth emphasizing that the LBD GWAS’ [30, 31] we used, uniquely included autopsy-confirmed case-ascertainment, hence the observed associations reflect true LBD rather than a diagnosis based on clinical manifestation. Importantly, we did observe a protective, albeit relatively attenuated, effect of lower CETP concentration decreasing the risk of LBD in non *APOE-*ε4 carriers when weighting by Apo-A1: OR 0.65 95%CI 0.52; 0.80 (*p*-value 4.11 × 10⁻⁵). This association did not reach statistical significance in the Apo-B or CETP concentration weighted analyses and therefore requires further confirmation. While we did not have access to genetic data on vascular dementia (VD), we were able to show that decreased plasma concentrations of CETP protect against small vessel stroke, which is the primary risk factor for VD. Providing further guidance to the current efforts expanding the CETP inhibitor obicetrapib for treatment of dementia. While we did observed replicated effects of lower CETP concentration on larger brain volume, we did not observe a similarly concordant effect of lower CETP on white matter hyperintensity volume or circulation total tau. Potentially, this reflects a lack of *APOE*4 stratification [52], lack of data on regional volumes [53], or simply distinct pathways with brain volume more closely relating to plaque forming. Additionally, we observed a protective effect of lower CETP against dementia in PD, which provides further evidence for CETP involvement in *APOE4* driven phenotypes.

The MR analyses performed in this study are protected against bias due to pre-translational horizontal pleiotropy by combining a model selection framework (providing a data-driven choice between IVW and MR-Egger MR methods) with removal of potential pleiotropic variants based on contributions to the leverage or heterogeneity statistics. Furthermore, our analysis of the effect of lower CETP concentration on blood lipids and CVD outcomes are in-line with findings from CETP inhibitor trials [5, 6], strongly suggesting the presented MR findings are protected against pre-translational pleiotropy. Given that the effects of CETP on dementia are anticipated to follow from its effect on lipid metabolism, the positive control CVD effects suggest that the associations with dementia traits may be similarly robust to pre-translational horizontal pleiotropy bias. Furthermore, our analysis was protected against bias due to potential weak-instruments and winner’s curse by selecting genetic variants strongly related with CETP concentration, using a F-statistic threshold of 24 or larger. Second, the GWAS on CETP concentration was sourced from a single study conducted by Blauw et al. [32] , which has no sample overlap with the outcome GWAS ensuring that, on average, any potential for weak instrument bias acts towards a neutral effect direction [54].

As described previously [9, 11] a *cis-*MR analysis weighted by a downstream biomarker which is affected by the protein provides inference on the protein effect direction conditional on firm understanding on whether the protein increases or decreases biomarker concentration and/or activity. Such a biomarker weighted MR analysis does not provide evidence of potential downstream mediation effects. As such the presented Apo-A1 and Apo-B weighted *cis-*MR analysis represents directional tests of the effect of CETP *activity*, not of potential mediation by either apolipoprotein. Given the distinction between protein concentration and activity, the Apo-A1 and Apo-B weighted analyses – representing a combination of CETP concentration and activity rather than concentration alone, not only serve as partial replication, but also complements the *cis*-MR analysis based on CETP concentration. While the current analyses suggest that inhibition of CETP might protect against dementia, it does not provide information on the required dosage, timing and duration of CETP inhibition [8]. As such the reported effect estimates, while robust indicators of effect direction, are unlikely to reflect anticipated effect magnitudes of pharmacological inhibition of CETP. Our findings therefore call for careful re-analysis of existing (pre) clinical data on CETP inhibition, followed by potential *de novo* studies evaluating potential effects of CETP inhibition on dementia. In fact, our analyses are supported by recent studies in mice transgenic for both the human amyloid precursor protein (*APP*) gene, as well as *CETP*, showing accelerated AD progression concomitant with a 22% increase of cholesterol content in brain [28]. Moreover, administration of the CETPi evacetrapib rescued memory deficit in these *AAP/CETP*tg mice [28]. This beneficial change in cognition in evacetrapib treated mice correlated with both decreased LDL-C as well as increased HDL-C concentrations conferred by the CETPi.

In conclusion, our *cis*-MR recapitulated the beneficial on-target effects of lower CETP activity on blood lipids and CVD outcomes, mimicking the effect of pharmacologic CETP-inhibition. Consistent with known pathophysiology we expanded these analyses to show that lower CETP activity may elicit an *APOE4* dependent protective effect on Lewy body dementia and dementia associated with Parkinson’s disease. In conjunction with human data of loss-of-function alleles of CETP that protect against dementia in *APOE-*ε4 + carriers and preclinical data in a humanized rodent model of dementia that show rescue of cognition loss by a CETP-inhibition, these results suggest that CETP-inhibition might be repurposed for treatment of dementia in *APOE-*ε4 + carriers.

## Electronic supplementary material

Below is the link to the electronic supplementary material.

Supplementary Material 1

Supplementary Material 2

## Data Availability

The genetic data used for this analyses are available as Data 1 based on genomic build GRCh37. The individual GWAS data leveraged in this study can be accessed as followed: CETP concentration was available from Blauw et al. (n: 4,248 https://www.ahajournals.org/doi/full/10.1161/CIRCGEN.117.002034), Apo-A1, Apo-B, IDL-C, VLDL-C, remnant-C, from (n: 115,078, https://gwas.mrcieu.ac.uk/datasets, dataset: met-d), LDL-C, HDL-C, and TG from (n: 1,320,016, http://csg.sph.umich.edu/willer/public/glgc-lipids2021), lp[a] from (n: 361,194, http://www.nealelab.is/uk-biobank), systolic/diastolic blood pressure (n: 757,601, https://www.ebi.ac.uk/gwas/publications/30224653), brain volume, (n: 47,316, https://ctg.cncr.nl/software/summary_statistics), white matter hyperintensity volume (n: 42,310, https://www.ebi.ac.uk/gwas/publications/32358547), circulation total tau (n: 14,721, https://www.ebi.ac.uk/gwas/publications/35396452), coronary heart disease (cases: 181,522, total n: 1,165,690, https://www.ebi.ac.uk/gwas/publications/36474045), any stroke, ischemic stroke, small vessel stroke (any stroke cases: 110,182, total n: 1,614,080; any ischemic stroke cases: 86,668, total n: 1,590,566; small vessel stroke cases: 9,219, total n: 1,517,518, https://www.nature.com/articles/s41586-022-05165-3), atrial fibrillation (cases: 60,620, total n: 1,030,836 , https://www.ebi.ac.uk/gwas/publications/30061737), heart failure (cases: 115,150, total n: 1,665,481, https://www.ebi.ac.uk/gwas/publications/36376295), abdominal aortic aneurysm (cases: 8,163, total n: 1,164,713, https://www.globalbiobankmeta.org/), Lewy body dementia (cases: 2,981, total n: 6,618, https://www.ebi.ac.uk/gwas/publications/33589841), Lewy body stratified on APOE-ε4 status (positive cases: 1,180, positive total n: 1,837, negative cases: 1,286, negative total cases: 3,557, https://www.ebi.ac.uk/gwas/publications/35381062), Parkinson’s disease (cases: 56,306, total n: 14,056,306, https://www.thelancet.com/pdfs/journals/laneur/PIIS1474-4422(19)30320-5.pdf), dementia in Parkinson’s disease (cases: 263, total n: 3,923, https://pdgenetics.org/resources), multiple sclerosis (cases: 14,498, total n: 38,589, https://imsgc.net/), amyotrophic lateral sclerosis (cases: 15,156, total n: 41,398, https://www.nature.com/articles/ng.3622).

## Code availability

Analyses were conducted using Python v3.7.13 (for GNU Linux), Pandas v1.3.5, Numpy v1.21.6, bio-misc v0.1.4, and matplotlib v3.4.3. The code underpinning the presented results can be accessed here: https://gitlab.com/SchmidtAF/cetp_dementia .
