# Correction: Lower activity of cholesteryl ester transfer protein (CETP) and the risk of dementia: a mendelian randomization analysis

**DOI:** 10.1186/s13195-024-01639-w

**Published:** 2024-12-27

**Authors:** Amand F. Schmidt, Michael H. Davidson, Marc Ditmarsch, John J. Kastelein, Chris Finan

**Affiliations:** 1 Institute of Cardiovascular Science, Faculty of Population Health, University College London, 69-75 Chenies Mews, London, WC1E 6HX UK; 2 UCL British Heart Foundation Research Accelerator, 69-75 Chenies Mews, London, WC1E 6HX UK; 3 Department of Cardiology, Amsterdam Cardiovascular Sciences, Amsterdam University Medical Centres, University of Amsterdam, Amsterdam UMC, locatie AMC Postbus 22660, Amsterdam Zuidoost, 1100 DD The Netherlands; 4 Department of Cardiology, Division Heart and Lungs, University Medical Center Utrecht, Utrecht University, Heidelberglaan 100, Utrecht, 3584 CX The Netherlands; 5 Pritzker School of Medicine, University of Chicago, 5801 S Ellis Ave, Chicago, IL 60637 USA; 6 NewAmsterdam Pharma B.V, Gooimeer 2-35, Naarden, 1411 DC Netherlands; 7 Department of Vascular Medicine, Amsterdam Cardiovascular Sciences, Amsterdam University Medical Centres, University of Amsterdam, Amsterdam UMC, locatie AMC Postbus 22660, Amsterdam Zuidoost, 1100 DD The Netherlands

**Correction: Alz Res Therapy 16**,** 228 (2024)**

10.1186/s13195-024-01594-6

Following publication of the original article [1], an important textual error was noticed and corrected in the fourth paragraph of Background section.

The current wording erroneously states that CETP inhibitors decreases Apo-E concentration:

However, all CETP inhibitors, including the novel CETP-inhibitor obicetrapib, lowers plasma concentrations of apolipoprotein-E (Apo-E) [4] which is a known risk factor for dementia, in particular for Alzheimer’s disease (AD).

Whereas the cited reference [4] actually shows an increasing effect. The correct wording should read:

However, all CETP inhibitors, including the novel CETP-inhibitor obicetrapib, increase plasma concentrations of apolipoprotein-E (Apo-E) [4], which is associated with decreased risk of dementia, in particular for Alzheimer’s disease (AD).

The original article [1] has been updated.
